# Associations Between Non-communicable Diseases and Obstetric Complications: A Retrospective Records Review at a Tertiary Referral Hospital in Uganda

**DOI:** 10.7759/cureus.71156

**Published:** 2024-10-09

**Authors:** Leevan Tibaijuka, Joseph Ngonzi, Jean-Pierre Van Geertruyden, Asiphas Owaraganise, Lisa M Bebell, Musa Kayondo, Francis Bajunirwe, Yarine F Tornes, Yves Jacquemyn, Adeline A Boatin

**Affiliations:** 1 Obstetrics and Gynecology, Mbarara University of Science and Technology, Mbarara, UGA; 2 Global Health Institute, Department of Family Medicine and Population Health, Faculty of Medicine and Health Sciences, University of Antwerp, Antwerp, BEL; 3 Clinical Division, Infectious Diseases Research Collaboration, Kampala, UGA; 4 Medicine, Massachusetts General Hospital, Harvard Medical School, Boston, USA; 5 Community Health, Mbarara University of Science and Technology, Mbarara, UGA; 6 Obstetrics and Gynecology, Antwerp University Hospital (UZA), Antwerp, BEL; 7 Obstetrics and Gynecology, Massachusetts General Hospital, Harvard Medical School, Boston, USA

**Keywords:** maternal health, maternal morbidity, non-communicable diseases, obstetric complications, sub-saharan africa

## Abstract

Objective: Non-communicable diseases (NCDs) increasingly contribute to maternal morbidity and mortality. We determined the association between NCDs and obstetric complications at Mbarara Regional Referral Hospital (MRRH) in southwestern Uganda.

Methods: In this retrospective records review, we randomly selected records of women admitted for delivery at MRRH each month from January to December 2022, and extracted their socio-demographic and clinical histories. We defined a history of NCDs as chronic hypertension, pre-gestational diabetes, cardiac disease, anemia, or asthma. We performed a multivariate robust Poisson regression analysis to assess the association between NCDs and obstetric complications, including preeclampsia, gestational diabetes, venous thromboembolic disease, obstetric hemorrhage, and preterm labor. Models were adjusted for maternal age, gravidity, referral status, employment status, and human immunodeficiency virus (HIV) serostatus.

Results: We extracted data for 2,336 women with a mean age of 26±5.9 years. At least one NCD was present in 6.4% (n=149) of the patients, including anemia (n=77, 3.3%), chronic hypertension (n=35, 1.5%), pre-gestational diabetes (n=16, 0.7%), asthma (n=9, 0.4%), and cardiac disease (n=6, 0.3%). Overall, 542 (23.2%) women had obstetric complications, including preeclampsia (n=265, 11.3%), preterm labor (n=67, 2.9%), placental abruption (n=29, 1.2%), postpartum hemorrhage (PPH) (n=54, 2.3%), and gestational diabetes (n=5, 0.2%). Women with NCDs had an increased likelihood of having an obstetric complication compared to women without (overall proportion 33.6% vs 22.5% respectively); adjusted prevalence ratio (aPR) was 1.8 (95% CI: 1.4-2.3) overall, 1.8 (95%CI: 1.2-2.8) for preeclampsia, 12.0 (95%CI: 2.0-72.7) for gestational diabetes, 6.0 (95%CI: 1.3-27.1) for deep venous thrombosis, 4.4 (95%CI: 1.5-12.6) for placenta abruption, and 4.3 (95%CI: 2.2-8.3) for PPH.

Conclusions: We found that NCDs were associated with a nearly two-fold increase in the risk of obstetric complications. Our findings highlight the need for further research to understand the impact of this risk, particularly on maternal and fetal outcomes. Additionally, these findings suggest strengthened NCD surveillance, as a means of increasing preparedness, and management of potential obstetric complications among pregnant women in Uganda.

## Introduction

Globally, non-communicable diseases (NCDs) are a substantial contributor to morbidity and mortality, accounting for 15% of maternal deaths globally and 19% of deaths in sub-Saharan Africa (SSA) [1,2]. Thus, although NCDs are considered an indirect cause of maternal mortality, their contribution represents about half of the deaths caused by postpartum hemorrhage, the leading cause of direct obstetric-related deaths [2].

SSA is currently undergoing an epidemiological transition, with an increasing incidence and prevalence of NCD-related morbidity and mortality [3]. NCDs during pregnancy may significantly aggravate obstetric conditions or may be aggravated by the physiological effects of pregnancy [4-6]. Thus, the increase in NCD seen in the region may also contribute to increasing rates of obstetric complications as suggested by a limited amount of research on it in SSA [3,6,7], and a larger body of work demonstrating an association with increased prevalence of NCDs and increased rates of obstetric complications and poor maternal-fetal outcomes.

However, despite the increasing rates of NCD and the potential link to increasing obstetric complications, there has been insufficient attention in low- and middle-income countries (LMICs) overall to NCDs, as evidenced by limited screening and diagnosis, management, and follow-up after the pregnancy period [3,6], and a paucity of data on the impact of NCDs in pregnancy on maternal health and pregnancy outcomes in LMICs including within SSA. Furthermore, the data currently available on NCDs in pregnancy in SSA are limited by scarce and fragmented data, which makes it difficult to understand the scope and burden of the problem, inadequate surveillance systems, underreporting, limited information on risk factors, and maternal and fetal outcomes [6,8]. The available data are also limited by gaps in healthcare provider training and capacity to diagnose and manage NCDs in pregnancy, inadequate policies and guidelines, and limited resources and funding that hinder efforts to address NCDs in pregnancy [6,7,9,10]. Consequently, NCDs have not yet received adequate attention from the key policy and strategic frameworks of national and international maternal health organizations [11,12].

In Uganda, there is a significant lack of national data regarding NCDs in general, with a few small, geographically localized, hospital and population-based epidemiological studies on independent NCDs, such as hypertension (HTN), diabetes mellitus (DM), rheumatic heart disease (RHD), and their risk factors among others [13-18]. In addition, only a few studies have explored NCDs in pregnancy in Uganda, citing a significant number of these conditions contributing to maternal morbidity, yet with limited care facilities [6,19]. Therefore, this study aimed to determine the association between NCDs and obstetric complications among women admitted to a large regional health facility in Uganda. 

## Materials and methods

Study population and setting

This study was conducted at Mbarara Regional Referral Hospital (MRRH), a government-funded public referral and teaching hospital affiliated with the Mbarara University of Science and Technology. It is located in southwestern Uganda, an area with a population of nine million people across nearly a dozen districts. Patients also present to MRRH from neighboring countries to seek medical care. The MRRH maternity ward registers approximately 8,000-10,000 deliveries annually and has a maternal mortality rate of 375 per 100,000 live births in the five years from 2015 to 2019 and a perinatal mortality rate of 33 per 1000 live births in 2020 [20,21]. The obstetrics department has 14 obstetricians and gynecologists, 38 postgraduate students, interns, nurses, and midwives.

MRRH provides a wide range of preventive and curative care, serves as a critical referral center, and houses a high-risk ward dedicated to managing mothers with complicated pregnancies, making it a crucial destination for those with life-threatening medical and obstetric conditions. Furthermore, it has the only functional adult intensive care unit in the region that capable of providing life support. The hospital also houses a functional neonatal intensive care unit that addresses the needs of newborns with complications [22].

Study design

We conducted a retrospective review of records. The study was approved by the Research Ethics Committee of Mbarara University of Science and Technology (approval number: MUST-2021-143) and the Uganda National Council for Science and Technology (approval number: HS1734ES). We identified all admissions (including antepartum, intrapartum, and postpartum) to the maternity ward from January 2022 to December 2022 at the MRRH. We then selected a random sample of 200 admissions from each month of 2022 using in-patient numbers from the obstetrics admissions register and obtained their records for chart abstraction. Chart abstraction was performed by trained research assistants using a Research Electronic Data Capture (REDCap) electronic data capture tools hosted at MRRH [23].

Data collection

Chart abstracted data included demographic information (age, parity, district of origin), medical (including NCDs) and obstetric history, referral history, and diagnoses made during admission including NCDs.

The primary outcome was the presence of any one or more obstetric complications including preeclampsia/eclampsia, gestational DM, venous thrombo-embolic (VTE) disease, placental abruption, placenta previa, preterm premature rupture of membranes (PPROM), preterm labor, severe oligohydramnios, and postpartum hemorrhage. We considered one or more of these conditions as present if there was documentation of the condition in the medical record. At the maternity ward of MRRH, these conditions are routinely diagnosed following the following criteria: gestational HTN was defined as new-onset HTN without proteinuria [24,25]. Preeclampsia was defined as new onset HTN with proteinuria (≥ 2+ protein) or without proteinuria but with severe features of pre-eclampsia including severe hypertension (systolic blood pressure ≥160 mmHg or diastolic ≥110 mmHg), persistent epigastric pain, persistent headache, visual changes, elevated creatinine, and elevated liver transaminases [24,25]. Eclampsia was defined as the presence of grand mal seizures in participants with signs and symptoms of pre-eclampsia [26]. Gestational DM was defined as glucose intolerance that was first detected during the second or third trimester of the current pregnancy [27-29]. Deep-vein thrombosis (DVT) was defined as the presence of a blood clot in the lower extremities following a compression Doppler ultrasonography [30,31], and pulmonary embolism (PE) was defined as the presence of dyspnea, abrupt onset of chest pain, cough and other signs in participants with a diagnosis of DVT [30-32].

The primary predictor was the presence of NCD. We considered women to have an NCD if their medical chart included documentation by a clinician of any of the following diagnoses: chronic HTN, pre-gestational DM, cardiac disease, anemia, epilepsy, and asthma. Documentation of NCDs in the medical record is typically made after a self-report from a patient, or if there is evidence of prior diagnostic investigations or ongoing treatment related to these conditions. The other characteristics collected included maternal age, marital status, gravidity, employment status, level of education, referral status, HIV serostatus, smoking, and alcohol use. Anemia was defined as hemoglobin estimation of <11.0 g/dL as noted from the participant’s chart [33-35]. HIV diagnosis was made per clinical chart review or self-reported HIV serostatus. 

Sample size calculation and sampling

Approximately 8,000-10,000 women deliver annually at MRRH. We estimated the sample size based on the prevalence of maternal medical comorbidities reported by the high-dependence unit of Mulago National Referral Hospital based in Uganda’s capital city, Kampala, which was 20.7% [19]. Considering a 95% confidence interval (CI) and a power of 80%, we estimated we would need a sample size of 2,400 women (200 women each month for 12 months) from the obstetrics admissions register at MRRH to estimate the prevalence of NCDs in this population. We obtained monthly in-patient numbers from the obstetric admissions registers of MRRH for each month for the year 2022 into an Excel sheet (Microsoft Corporation, Redmond, Washington, United States) and randomly selected a sample of 200 patient charts each month.

Data analysis

Data were exported to STATA 17 (2021; StataCorp LLC, College Station, Texas, United States) for cleaning and analysis. We compared the participant characteristics among women with and without NCDs using t-tests for continuous variables, and Chi-square test and Fisher's exact test for categorical variables. We described the NCDs and obstetric complications as frequencies. We performed a multivariate robust modified Poisson regression analysis to assess the associations between NCDs and obstetric complications (as defined above). We determined the associations between NCDs and a composite outcome of one or more obstetric complications (including preeclampsia, gestational DM, VTE, antepartum hemorrhage, preterm labor, preterm premature rupture of membranes, and postpartum hemorrhage), as well as each individual obstetric complication. Models were adjusted for maternal age, gravidity, referral status, employment status, and HIV serostatus. To assess collinearity in our multivariate models, we employed the variance inflation factor (VIF) method, considering VIF >5 as indicative of collinearity. We considered a p-value of <0.05 to be statistically significant.

## Results

There were 8,571 obstetric admissions at MRRH from January 2022 to December 2022. Of these, we randomly selected 2400 patient charts for inclusion in the study. Of these, we excluded 64 records comprising 43 (67%) because the clinical charts could not be located and 21 (33%) because the charts were missing information on more than half of the exposure or outcome variables.

Characteristics of study participants

The mean age of the participants was 26.5±5.9 years. Most were married (98.6%), had two to four pregnancies (51.1%), had attended at least one antenatal care visit (99.1%), and had a gestation age between 37 and 42 weeks (64.2%). Participants with NCDs were significantly older (28.6±7.0 years versus 26.4±5.8 years), were more likely to have been referred/transferred from another health facility to MRRH (54.4% versus 26.9%), attended <4 antenatal care (ANC) visits (42.3% versus 26.2%) and had >4 pregnancies (28.2% versus 14.8%) compared to those without NCDs (Table 1).

**Table 1:** Characteristics of the study sample *p<0.05 Data given as n (%) except for age which is given as mean±SD

Variables	Non-communicable diseases			P-value
	Total (n=2,336), n (%)	Yes (n=149), n (%)	No (2,187), n (%)	
Age (years), Mean±SD	26.±5.9	28.6±7.0	26.4±5.8	<0.001*
Maternal age category (years)				<0.001*
<20	213 (9.1%)	8 (5.4%)	205 (9.4%)	
20-34	1,839 (78.7%)	107 (71.8%)	1,732 (79.2%)	
>34	284 (12.2%)	34 (22.8%)	250 (11.4%)	
Reside in Mbarara	1,440 (62.7%)	56 (37.6%)	1,584 (63.3%)	<0.01*
Employed	1,055 (60.9%)	76 (69.1%)	979 (60.4%)	0.093
Married	1,895 (98.6%)	118 (96.7%)	1,777 (98.8%)	0.057
Referred	670 (28.7%)	81 (54.4%)	589 (26.9%)	<0.001*
HIV seropositive	207 (10.6%)	10 (9.1%)	197 (10.7%)	0.590
Gravidity				<0.001*
Primigravida	776 (33.2%)	46 (30.9%)	730 (33.4%)	
Multigravida	1,194 (51.1%)	61 (40.9%)	1,133 (51.8%)	
Grand multigravida	366 (15.7%)	42 (28.2%)	324 (14.8%)	
Antenatal care visits				<0.001*
<4	635 (27.2%)	63 (42.3%)	572 (26.2%)	
≥4	1,701 (72.8%)	86 (57.7%)	1,615 (73.8%)	
Gestational age (weeks)				<0.001*
<26	74 (3.8%)	28 (25.0%)	46 (2.5%)	
26-<37	487 (25.3%)	56 (50.0%)	431 (23.8%)	
37-42	1,236 (64.2%)	26 (23.2%)	1,210 (66.7%)	
>42	129 (6.7%)	2 (1.8%)	127 (7.0%)	

NCDs and obstetric complications

A total of 149 (n=6.4%) admissions had chart documentation of an NCD. These included anemia in 77 (3.3%), chronic HTN in 35 (1.5%), pre-gestational DM in 16 (0.7%), asthma in nine (0.4%), and cardiac disease in 6 (0.3%). Overall, 542 (23.2%) women had one or more obstetric complications, including pre-eclampsia (n=265, 11.3%), preterm labor (n=67, 2.9%), placenta abruption (n=29, 1.2%), PPH (n=54, 2.3%), and gestational DM (n=5, 0.2%). Obstetric complications were more common among women with NCDs (33.6%, 50/149) compared to those without NCDs (22.5%, 492/2187), p<0.01.

Associations of NCDs with obstetric complications

Women with NCDs had an increased likelihood of having one or more obstetric complications (adjusted prevalence ratio (aPR) 1.77, 95%CI: 1.35-2.32), pre-eclampsia (aPR 1.83, 95%CI: 1.21-2.76), gestational DM (aPR 11.99, 95%CI: 1.98-72.71), DVT (aPR 6.02, 95%CI: 1.34-27.07), placenta abruption (aPR 4.37, 95%CI: 1.51-12.61), and post-partum hemorrhage (aPR 4.26, 95%CI: 2.19-8.29) (Table 2). When each NCD was compared with one or more obstetric complications, chronic HTN (aPR 1.97, 95%CI: 1.33-2.93) and pre-gestational DM (aPR 1.24, 95%CI: 1.01-2.48) were independently associated with one or more obstetric complications.

**Table 2:** Associations of NCDs with obstetric complications in the study sample cPR: crude prevalence ratio; aPR: adjusted prevalence ratio; PPROM: Preterm premature rupture of membranes; NCD: non-communicable disease ^#^Each model was adjusted for maternal age, gravidity, referral status, employment status, and HIV serostatus; *p < 0.05

Outcomes	NCDs		Unadjusted analysis		Multivariate analysis	
	Yes (n=149)	No (n=2,187)	Crude PR (95% CI)	P-value	Adjusted PR (95% CI)^#^	P-value
	n (%)	n (%)				
Primary outcome						
Any one or more obstetric complications	50 (33.6)	492 (22.5)	1.49 (1.17-1.89)	0.001*	1.77 (1.35-2.32)	<0.001*
Secondary outcomes						
Pre-eclampsia/Eclampsia	28 (18.8)	237 (10.8)	1.73 (1.22-2.47)	0.002*	1.83 (1.21-2.76)	0.004*
Gestational diabetes mellitus	2 (1.3)	3 (0.1)	9.79 (1.65-58.13)	0.012*	11.9 (1.98-72.71)	0.007*
Deep venous thrombosis	2 (1.3)	4 (0.2)	7.34 (1.35-39.76)	0.021*	6.02 (1.34-27.07)	0.019*
Pulmonary embolism	2 (1.3)	2 (0.1)	14.68 (2.1-103.5)	0.007*	8.69 (0.88-86.22)	0.065
Placental abruption	5 (3.4)	24 (1.1)	3.06 (1.18-7.90)	0.021*	4.37 (1.51-12.61)	0.006*
Placenta previa	1 (0.7)	28 (1.3)	0.52 (0.07-3.83)	0.524	0.27 (0.035-2.04)	0.203
Postpartum haemorrhage	12 (8.1)	42 (1.9)	4.19 (2.26-7.79)	<0.001*	4.26 (2.19-8.29)	<0.001*
PPROM	1 (0.7)	71 (3.2)	0.21 (0.03-1.48)	0.116	0.23 (0.03-1.67)	0.148
Preterm labor	1 (0.7)	66 (3.0)	0.22 (0.03-1.59)	0.134	0.27 (0.04-2.04)	0.203
Severe oligohydramnios	2 (1.3)	27 (1.2)	1.09 (0.26-4.53)	0.909	1.64 (0.39-6.79)	0.492

## Discussion

This cross-sectional study assessed the association of NCDs with obstetric complications at a tertiary referral hospital in a low-resource setting in southwestern Uganda. Overall, we found obstetric complications were nearly two-fold more common among women with NCDs compared to those without. Women with NCDs had an increased risk of having one or more obstetric complications and were also at increased risk of pre-eclampsia, gestational DM, DVT, placenta abruption, and postpartum hemorrhage. These findings highlight the need to strengthen surveillance and management of NCDs and hospital preparedness for screening and managing the associated obstetric complications, especially in low-resource settings where access to obstetric medicine specialists and advanced obstetric care may be limited.

The NCD prevalence of 6.4% found in our study was lower than what is documented in other low-resource settings both among obstetric and the general reproductive-age populations [6,36,37]. In Pakistan, the prevalence of NCDs among obstetric admissions was 88.4% [38]. Studies from India [39], Nepal [40], Bangladesh [41], and Kenya [42] among women of reproductive age reported a prevalence of 77.0%, 39.0%, 34.6%, and 15.9%, respectively. There is, however, scanty epidemiological data regarding NCDs in sub-Saharan Africans in the general population and worse still in pregnancy [6,9,36]. Our low prevalence of NCDs could be explained by the under-diagnosis of these conditions as the population we enrolled included mainly women with more severe NCDs referred to our study site, which serves as the main tertiary referral hospital in southwestern Uganda, while the asymptomatic (non-severe) cases were probably not enrolled. The underdiagnosis could also be explained by the low investigative capacity and probable underreporting, which may be experienced in low-resource settings like ours. In particular, for Uganda, the prevalence found in our study was substantially lower than close to 21% of what was documented at Mulago National Referral Hospital [19]. This could be explained by the fact that Mulago is the national referral hospital and probably included patients that are sicker in general including those with pregnancy-related NCDs (like preeclampsia/eclampsia), referred from across the country, and also has a different more urban population.

Despite the lower overall prevalence in the current study, NCDs were associated with a nearly two-fold increased risk of any obstetric complications, with the risk even higher among individual obstetric complications. Medical conditions, specifically NCDs, have been shown to affect pregnancy and increase the risk of obstetric complications in prior studies in LMICs; this further contributes to the morbidity and mortality of these women [6,43]. Given that most women with NCDs were referred in from the peripheral facilities, this further highlights the issue of underdiagnosis of NCDs with the cases identified and referred being the more severe cases. It is also likely that management of NCDs like DM, HTN, cardiac disease, etc., may have been sub-optimal in our low-resource setting. Optimal management of these NCDs in pregnancy requires a ready, well-organized, and well-resourced setting; this may be lacking in many LMICs [6-8]. 

We found a high prevalence of one or more obstetric complications of 23.2%. These were more common among women with NCDs (33.6%) compared to those without NCDs (22.5%). The most common obstetric complications were preeclampsia (11.3%), preterm labor (2.9%), and PPH (2.3%). Our findings are similar to those from prior studies that noted preeclampsia and PPH as common obstetric complications [20,44], which are also the leading causes of maternal and fetal morbidity and mortality in our setting. 

Obstetric conditions like gestational DM, pre-eclampsia, preterm birth, and placental abruption may equally contribute to the development of cardiovascular diseases later in life [45-48]. In our study population, the largest group of individuals with NCDs consisted of women who had been pregnant more than once. It's possible that their previous pregnancy complications contributed to the development of NCDs before their current pregnancy. This highlights the significant linkage between NCDs and obstetric conditions and calls for strengthening the timely identification and management of both NCDs and obstetric complications. It is still unclear whether obstetric complications predispose, exacerbate, or cause NCDs [48]; however, some mechanisms have been suggested to link NCDs to obstetric complications including inflammation and oxidative stress, endothelial dysfunction, insulin resistance, metabolic changes, and epigenetic modifications [49-53]. This calls for a deeper understanding of these pathways, considering the shared risk factors playing a role in these mechanisms.

Much as this is beyond the scope of our study, the multi-morbidity associated with the combination of NCDs and obstetric complications is also likely to increase adverse maternal-fetal outcomes, contributing to mortality and poor quality of life. A comprehensive approach encompassing multiple strategies is warranted, especially focusing on the prevention of NCDs in reproductive-age women in primary healthcare, including health promotion, community-based programs, and deliberate cost-effective policies targeting the general population and those at increased risk, especially the women likely to suffer a double burden of NCDs and pregnancy-related comorbidities [54,55].

Strengths and limitations of the study

The strength of our study was that it provides information about the neglected global concern of NCDs in pregnancy and how they relate to the emergency obstetric complications in low-resource settings like ours where there is reported increasing maternal ill-health associated with NCDs [6]. However, our study was not without limitations. First, our study was hospital-based and only included obstetric admissions at a single tertiary referral hospital (excluding women in the antenatal and post-natal clinics). Secondly, NCD diagnosis was largely based on self-report (or evidence of prior diagnostic investigations or ongoing treatment related to these conditions) and many conditions like chronic HTN, DM, and cardiac disease may have been asymptomatic, particularly when not severe, hence the likelihood of underdiagnosis of some conditions, and this may therefore have underestimated the number of women with NCDs. However, our study population of obstetric admissions represented a population in whom assessment of obstetric complications was possible. Thirdly, our findings are from a single hospital and may not represent the entire region and community. However, the biggest contributor to the NCDs was women referred from other peripheral health units to our study site, a tertiary referral hospital. Fourthly, given the cross-sectional nature of our study, causality may not be inferred, but, given that the NCDs reported in this study were documented as pre-pregnancy, they may indeed explain the associations with obstetric complications. Lastly, we noted a significant amount of missing data for some study variables since the source of the data was the patient charts and also noted some missing charts (1.8%). It is therefore possible that these were not randomly missing, which could impact the overall findings. 

## Conclusions

Our study found an association between NCDs and a higher risk of obstetric complications during pregnancy; women with NCDs were nearly twice as likely to experience any of these complications. Future research is needed to explore the maternal-fetal outcomes among women with NCDs, especially those with multi-morbidity (NCDs and obstetric complications) in similar low-resource settings. Our findings also suggest the need for improved screening and identification of NCDs to identify women potentially at risk of obstetric complications who may benefit from higher levels of intrapartum care. This is particularly important in resource-limited settings where access to obstetric medicine specialists and advanced obstetric management may be more limited.
